# PP09 Automated Study Selection Flowchart: Direct_Flow

**DOI:** 10.1017/S0266462325102298

**Published:** 2025-12-29

**Authors:** Rafael Leite Pacheco, Carolina Latorraca, Patrícia do Carmo Silva Parreira, Ana Luiza Cabrera Martimbianco, Verônica Colpani, sabela Porto Toledo, Roberta Borges Silva, Camila Monteiro Cruz, Cecília Menezes Farinasso, Rachel Riera

## Abstract

**Introduction:**

A fundamental stage of any evidence synthesis is the description of the study selection process. This description is ideally represented through flowcharts, which should contain the results of the searches, the number of studies eliminated in the initial phase of title and abstract screening, the number of studies excluded in the subsequent phase of full-text review, and the final number of studies included.

**Methods:**

This is a descriptive study of the direct_flow program, developed using the ado programming language within Stata software. This program was developed in the context of two projects conducted by the Center of Health Technology Assessment, Hospital Sírio-Libanês (NATS/NEv-HSL), São Paulo, Brazil and various governmental health organizations, including the Ministry of Health, the National Supplementary Health Agency, and the National Council of Justice, as part of the Support Program for Institutional Development of the Unified Health System (PROADI-SUS). The direct_flow program was used in both projects and aims to enhance the efficiency and consistency of evidence summaries by automating flowchart generation.

**Results:**

The direct_flow program operates using pre-programed templates, which can be chosen by the researcher. Eleven templates were created: seven tailored for systematic reviews and four for overviews of systematic reviews. The researcher needs to specify the number of references at each stage of the selection process through standardized commands. The program automatically populates the flowchart boxes and generates high resolution figures. All templates are available in Portuguese and English and can be downloaded via Stata. More details and access to the templates can be found at https://rlpacheco.github.io/direct_flow/.

**Conclusions:**

Drawing up evidence summaries can be complex and time consuming. Automating the generation of figures and other steps using direct_flow can reduce workload, while ensuring high quality and consistency across different syntheses. Although developed as part of two projects funded by PROADI-SUS, direct_flow is freely available for all HTA researchers.

